# Hearing assessment in transfusion dependent beta-thalassemia children on oral iron chelating agent

**DOI:** 10.1186/s12887-025-06116-3

**Published:** 2025-10-03

**Authors:** Wafaa E. I. Mohamed, Marwa Waheed Tolba, Yara Khalid Abuelfadl, Abeer Mohamed Elgendy, Heba G. A. Ali

**Affiliations:** 1 Pediatric Haematology Oncology and BMT Unit. Faculty of Medicine, Ain Shams University, Cairo, Egypt; 2 Audiology Unit, Otorhinolaryngology Department, Faculty of Medicine, Ain Shams University, Cairo, Egypt

**Keywords:** Paediatric, Hearing loss, Thalassemia, Iron-chelation

## Abstract

**Background:**

Hearing deficit is one of the side effects of 1st generation iron chelators in β-thalassemia, however the risk of hearing deficits following 2nd generation iron chelators is not well known.

**Aim:**

To assess hearing status of Transfusion Dependent β-thalassemia children on oral iron chelating agents and detect risk factors for hearing impairment.

**Methods:**

This is a cross-sectional study recruited sixty children and adolescent with confirmed diagnosis of transfusion dependent β-thalassemia. Demographic and clinical characteristics collected, audiological testing were performed by the same audiologist using the same equipment for all patients including tympanometry, pure tone audiometry, speech audiometry, transient evoked otoacoustic emissions and distortion product otoacoustic emissions.

**Results:**

Recruited children and adolescents with transfusion dependent β-thalassemia were 32 (53.3%) boys and 28 (46.7%) girls and their mean age was 11.34 ± 3.08, majority of patients 48 (80%) were on single Deferasirox. Our study revealed that among the 60 children evaluated, 16.6% exhibited some form of hearing loss. Sensorineural hearing loss (SNHL) was observed in 6.6% of the participants, while 10% had conductive hearing loss (CHL). Bilateral SNHL in 5% and bilateral CHL in 8.3% of all the cases. Hearing impairment was mild in nature, but predominantly affected high-frequency ranges, the most affected frequencies being 4000 Hz and 8000 Hz. There was no significant difference between studied thalassemia children with and without hearing impairment regarding gender, age at study entry, age at diagnosis, duration of disease and duration or dose of chelating agent (*P* > 0.05). Our study revealed significant difference between studied thalassemia children with and without hearing impairment regarding age of starting blood transfusion (*p*-value = 0.024), affected patients started blood transfusion at older age, also statistically significant difference in both groups regarding median serum ferritin was found (*p*-value = 0.028), lower levels were found in affected patients.

**Conclusion:**

No significant effect of using oral iron chelation drugs was observed on frequency and type of hearing loss among the studied patients but instead the age at starting regular blood transfusion did. Screening of such group of patients for hearing impairment at diagnosis and at regular periods is recommended.

## Introduction

Β-thalassemia is a common inherited congenital disorder of hemoglobin production, which results in hemolytic anemia as well as multi-organ involvement [1]. Almost 60,000 children with β -thalassemia are born worldwide yearly. Carriers are estimated to be around 1.5% of the global population) [2].

Management strategies for patients with thalassemia major consists of regular blood transfusions and iron chelating agents as well as management of secondary complications resulting from iron overload. Three main iron chelating medications are present: Deferoxamine (DFO), Deferiprone and Deferasirox. Many adverse effects are associated with iron chelators including ophthalmic, neurologic and renal complications, hearing loss, and infection [3].

The prevalence of hearing loss was found to be around 32.3% among patients with thalassemia major [4]. Different types of hearing loss were documented among patients with thalassemia major including conductive, sensorineural, and mixed hearing loss [4, 5].

Deferoxamine was considered the chelator of choice in the past. Although considered effective, Deferoxamine has to be given through the subcutaneous route for several hours and has a robust adverse effects including sensorineural hearing loss (SNHL) in 25% to 30% of patients [6]. As a result, other orally administered chelators as deferiprone and deferasirox (DFX) were developed. No recent data regarding the auditory adverse effects of these newer iron chelating agents. Hearing loss was reported to be up to 1.1% of patients in previous DFX clinical trials [7, 8]. Other studies reported mild hearing loss in nearly 45% of beta thalassemia patients receiving DFX [9, 10].

Regarding hearing evaluation in β-thalassemia, little data available for patients on DFX and deferiprone as majority of the studies were done on patients receiving Deferoxamine [11]. Moreover, no previous studies compared hearing loss rates between patients on the three iron chelators or studied hearing deficits among beta thalassemia patients who didn’t receive iron chelators.

This study aimed to assess hearing in Transfusion Dependent β-thalassemia (TDT) children on oral iron chelating agents for detection of its ototoxicity and the need for regular audiological assessment of β-thalassemia major patients for early detection and management, also to detect correlation between hearing status and other disease risk factors as severity of anaemia and iron overload.

### Study design

This is a cross-sectional study carried out at Paediatric Haematology Oncology and bone marrow transplant (BMT) Unit, Ain Shams University, Cairo, Egypt. All children and adolescents with confirmed diagnosis of TDT who are registered at the institutional database were screened to recruit only those who are older than 5 years till 18 years old. Patients with history of exposure to other ototoxic medications or patients who suffer from hearing loss 2ry to other known aetiologies of sensorineural hearing loss (prenatal, perinatal insults, postnatal insults, heredo-familial hearing loss and other systemic illness known to be associated with hearing loss) or inability to satisfactorily undergo audiologic testing in study were excluded. Eligible patients were enrolled till completion of the determined sample size. A sample size of at least 60 patients was needed, using the Epi info program for sample size calculation and reviewing results from the previous study by *Tartaglione and colleagues 2021* [4] showed that the overall prevalence rate of hearing impairment is 32.3% among beta-thalassemia patients, with a margin of error = 10% at a 90% confidence level.

The study was approved by the Research Ethics Committee, Faculty of Medicine, Ain Shams University (FMASU M S 489/2023) with Assurance No. FWA 00017585 in accordance with the Code of Ethics of the World Medical Association (Declaration of Helsinki) for experiments in humans, 2013.

### Study procedures

Before inclusion in the study, each caregiver gave a written signed and dated informed consent as well as an assent from each subject was obtained whenever applicable before study enrolment. Clinical characteristics including demographic (age at start of the study, age of diagnosis and gender), duration of the disease, symptoms of disease complications and symptoms of iron overload, starting age, frequency of blood transfusion and unit of blood received per year (Transfusion Index), chelation treatment including: age at starting, type, dose, duration and surgical history like splenectomy. Full otological history regarding: otalgia, otorrhoea, hearing impairment, itching, tinnitus, vertigo and others, in addition to inquiring about other risk factors for hearing loss were obtained.

Examination of patients included vital signs, anthropometric measurements, abdominal examination, cardiac, chest and neurological examination to detect any disease complications.

Otological examination: inspection of external ear, otoscopic examination of external auditory canal and tympanic membrane. Audiological testing was performed by the same audiologist using the same equipment for all patients including:

- *Tympanometry* to assess middle ear functions, tympanogram tracings are classified as type A (normal), type B (flat, clearly abnormal), and type C (indicating a significantly negative pressure in the middle ear, indicative of Eustachian tube dysfunction) [12]
- *Pure tone audiometry (PTA)*: was done in double-walled sound treated test booth: Air conduction thresholds using Supra-aural headphones were obtained for test frequencies from 250–8000 Hz. Bone conduction thresholds using bone vibrator at frequencies from 500- 4000 Hz. Mild degree of hearing loss is considered starting from thresholds > 25 dBHL [13]
- *Speech audiometry*: Speech reception threshold using Arabic bisyllabic words and Word recognition scores. Normal excellent scores are in the range from 88- 100% using Arabic Phonetically Balanced word lists [14]
- *Transient evoked otoacoustic emissions (TEOAE)*: were recorded in children with normal outer and middle ear. TEOAE was evoked by clicks at 80 dBpeSPL and were analysed at five frequency bands centred at 1, 1.4, 2, 2.8 and 4 kHz half octave bands with pass criteria ≥ 6 dB in at least 3 frequencies [15]
- *Distortion product otoacoustic emissions (DPOAE)*, were evoked by two simultaneously presented primary tones with two different frequencies (f1 and f2) at 1.22 ratio, at intensity levels of L1 = 65 dBSPL and L2 = 55 dBSPL measured across high frequencies of 6000, 8000, 10000. DPOAEs amplitudes (2f1-f2) and adjacent noise floors were averaged and ≥ 6 dB SNR considered a pass response [15, 16]

Laboratory investigations and imaging included initial Haemoglobin electrophoresis at diagnosis, mean level of complete blood count parameters (3 last consecutive results before blood transfusion), mean serum ferritin (the last 3 consecutive results).Echocardiography, Cardiac MRI T2* and Liver Iron Content (LIC) were included if available in patient’ files.

### Statistical analysis

Data analysis was done using Statistical Program for Social Science version 20.0 (SPSS Inc., Chicago, Illinois). Quantitative variables were presented in the form of mean and standard deviation for parametric data and in the form of median and inter-quartile range for non-parametric data. Qualitative variables were presented as number and percentage. Inferential analyses were done for quantitative variables using independent t-test in cases of two independent groups with parametric data and Mann Whitney U test in cases of two independent groups with non-parametric data. Inferential analyses were done for qualitative data using Chi square test for independent variables. While correlations were done using Pearson Correlation for numerical parametric data and using spearman rho test for numerical non-parametric and categorical data. A *p*-value < 0.05 was considered significant.

## Results

### Clinical characteristics of studied patients: (Table 1)

**Table 1:** Demographic and Clinical characteristics of studied patients

**Clinical characteristics**	TDT = 60
Age at presentation (years); Median (IQR)/Range	0.78 (0.5—2)/0.25–6
Age at study entry (years); Mean ± SD/Range	11.34 ± 3.08/6–18
Duration of illness (years); Mean ± SD/Range	9.86 ± 3.4/2–17.7
Gender; n (%)	
Male	32 (53.3%)
Female	28 (46.7%)
Type of disease; n (%)	
Thalassemia major	50 (83.3%)
Thalassemia intermedia	10 (16.7%)
Laboratory Characteristics,	
Mean haemoglobin(mg/dl) last year Mean ± SD/Range	7.79 ± 0.65/6.8–9.8
Mean serum ferritin (ng/dl) last year Median (IQR)/Range	1830.3 (997.5—2585.6)/431.3–11000
Imaging	
Cardiac MRI T2;n(%)	
No iron overload	27 (45%)
Iron overload	2 (3.3%)
Not done	31 (51.6%)
Cardiac T2*Value(ms); Mean ± SD/Range	31.12 ± 8.6/14.2–44.8
Degree of cardiac iron loading; n(%)	
No iron overload	27 (93.1%)
Mild	1 (3.4%)
Moderate	1 (3.4%)
Liver iron content; n(%)	
No iron overload	6 (3.6%)
Iron overload	23 (41.8%)
Not done	31 (51.6%)
Liver mean iron concentration (mg/g)); Median (IQR)/Range	7.23 (2.9—12.5)/1.97–28.7
Degree of hepatic iron loading; n(%)	
No iron overload	6 (20.6%)
Mild	3 (10.3%)
Moderate	17 (58.6%)
Severe	3 (10.3%)
Treatment Modalities	
Blood transfusion Median (IQR)/Range	
Starting age of blood transfusion(years)	0.75 (0.5—2)/0.25–9
Frequency of blood transfusion(/week**)**	3 (2—4)/1–6
Transfusion index per year (ml/kg/year**)**	160 (120—240)
Chelating agents	
Age at starting chelation (years)Median (IQR)/Range	2 (1.5—3)/1–10
Single deferasirox; n (%)	
Duration of deferasirox (years),Median (IQR)/Range	7 (5—9.2)/0–17
Total dose of deferasirox (mgs),Median (IQR)/Range	550 (360—900)/360–1080
Dose of deferasirox (mg/kg/day),Median (IQR)/Range	19.6 (13.8—25.7)/6.9–28.4
Single deferiprone, n (%)	1 (1.6%)
Combined deferiprone deferasirox; n (%)	11 (18.3%)
Total dose of deferiprone (mgs),Median (IQR)/Range	1000 (500—1000)/500–2500
Dose of deferiprone (mg/kg/day), Median (IQR)/Range	20.15 (15.35—31)/7.8–50

Sixty recruited children and adolescents with TDT (50 (83.3%) had β-thalassemia major and 10 (16.7%) had β-thalassemia intermedia who experienced progressive skeletal and facial changes as well as faltering growth so they were put on regular transfusion and dealt with as TDT afterwards), fifty-three (53.3%) were boys and 28 (46.7%) were girls and their mean age was 11.34 ± 3.08, the median age at diagnosis was 0.78 years**,** the mean duration of disease in years was 9.86 ± 3.4.

All patients included in the study are TDT, median starting age of blood transfusion in studied patients was 9 months with median transfusion index of 160 ml/kg/year.

The mean haemoglobin level of the participants was 7.79 ± 0.65 gm/dL and the mean serum ferritin was 1830.3 (997.5—2585.6) ng/mL. Results of MRI cardiac T2*were collected from patient's files showed only 29 (48.3%) out of 60 patients did it with mean of 31.12 ± 8.6(ms) with range 14.2–44.8(ms) (2 only showed signs of iron overload, one had mild iron overload and the other had moderate iron overload), also liver iron content measured by MRI with mean of 7.23 (2.9—12.5) (mg/g), 23 (41.8%) showed iron overload (17 (58.6%) had moderate iron overload,3 (10.3%) had severe overload and 3 (10.3%) showed mild iron overload).

Regarding chelating agents used, The majority of patients (*n* = 48; 80%) were on single deferasirox with median duration of 7 years (IQR 5–9.2) and median (IQR) dose of 19.6 (13.8–25.7) mg/kg/day with median total dose in mg/day of 550 (360—900) otherwise 18.3% of patients were on combined oral chelation (deferasirox and deferiprone). Median chelation duration of 8 (5.5—11) years was most frequent in the study sample.

### Audiological evaluaion of studied patients: (Tables 2 and 3)

**Table 2:** Percentage of patients with hearing loss; type, degree and affected frequencies

	**Total no. = 60**
Hearing loss	
No	50 (83.3%)
Yes	10 (16.7%)
Type of HL	
SNHL	4 (40.0%)
CHL	6 (60.0%)
Side of SNHL (Total no.4)	
Unilateral	1 (25%)
Bilateral	3 (75%)
Degree of SNHL (total no.7ears)	
Mild	7 (100%)
HL Frequency in SNHL	
High frequencies	3 (75%)
Combined low and high frequencies	1 (25%)
Side for CHL (Total no.6)	
Unilateral	1 (16.7%)
Bilateral	5 (83.3%)
Degree of CHL	
Mild	5 (83.3%)
Moderately severe	1 (16.7%)

^*^SNHL Sensorineural Hearing Loss, *CHL* Conductive hearing loss

**Table 3:** Comparison between total number of ears with and without hearing impairment regarding some demographic, clinical and management characteristics

		**Hearing affection**	**No hearing affection**	**Test value**	***P*** **-value**
		**No. = 16**	**No. = 29**		
Gender	**Male**	10 (62.5%)	15 (51.7%)	0.485^*^	0.486
	**Female**	6 (37.5%)	14 (48.3%)		
Age at study entry (years)	**Mean ± SD**	11.84 ± 2.36	11.21 ± 2.85	0.761•	0.451
	**Range**	8–17	6–17		
Age at diagnosis years	**Median (IQR)**	1 (0.54—3.25)	0.66 (0.5—1.4)	−1.597≠	0.110
	**Range**	0.33–6	0.25–6		
Duration of the disease years	**Mean ± SD**	9.95 ± 3.14	9.99 ± 2.67	−0.052•	0.958
	**Range**	4–16.42	5.4–16.25		
HB (g/dl)	**Mean ± SD**	7.81 ± 0.54	7.89 ± 0.69	−0.369•	0.714
	**Range**	6.8–8.77	6.8–9.8		
Mean serum ferritin ng/ml	**Median (IQR)**	1540.4 (652.55—2129.65)	1943.3 (1433.15—4167.7)	−2.196 ≠	0.028
	**Range**	431.3–3891	558.6–6194		
Age of starting blood transfusion/years	**Median (IQR)**	1.5 (0.54—4.5)	0.66 (0.33—1.4)	−2.265 ≠	0.024
	**Range**	0.4–9	0.25–6		
Frequency of blood transfusion	**Median (IQR)**	3.5 (2—4)	3 (3—4)	−0.447 ≠	0.655
	**Range**	2–6	2–6		
Transfusion index per year (ml/kg/year)	**Median (IQR)**	160 (120—240)	160 (120—160)	0.000 ≠	1.000
	**Range**	80–240	80–240		
Cardiac MRI T2 value (ms)	**Mean ± SD**	38.32 ± 4.78	29.82 ± 7.45	2.486•	0.026
	**Range**	32.5–44.8	21–41.2		
Liver iron content value (mg/g)	**Median (IQR)**	6.19 (2.65—7.74)	5.32 (2.6—13.76)	−0.434 ≠	0.664
	**Range**	2.27–11.6	1.97–28.7		
Deferiprone	**No**	14 (87.5%)	22 (75.9%)	0.873*	0.350
	**Yes**	2 (12.5%)	7 (24.1%)		
Deferasirox	**No**	16(0.0%)	29(0.0%)	-	-
	**Yes**	16 (100.0%)	29 (100.0%)		
Dose (/total mgs)	**Median (IQR)**	540 (405—720)	630 (500—1000)	−1.659 ≠	0.097
	**Range**	180–990	180–3000		
Duration of oral chelation years	**Median (IQR)**	7 (5—10)	9 (7—11)	−1.347 ≠	0.178
	**Range**	1–16	2—15		
Duration of (deferasirox) years	**Median (IQR)**	5.5 (5—8)	7 (6—9)	−1.099 ≠	0.272
	**Range**	1–14	0–14		

*HB* Hemoglobin, *MRI* Magnetic Resonance Imaging

*P*-value > 0.05: Non significant; *P*-value < 0.05: Significant; *P*-value < 0.01: Highly significant

^*^Chi-square test

•Independent t-test

≠ Mann–Whitney test

Regarding otological symptoms 23 (38.3%) out of 60 patients had otological symptoms (1(1.7%)patient had otalgia,1(1.7%)patient suffered from otorrhea, 19 (31.6%) patients suffered from diminution of hearing and 2 (3.3%) patients had tinnitus otherwise no patient suffered from vertigo or itching. None of the ten patients with hearing loss in the study complained of any difficulty in hearing or tinnitus and it was only on PTA that hearing loss was diagnosed.

Tympanometry revealed that majority of patients 42(71.2%) had Type A, 8(13.5%) had Type B and 9 patients (15.2%)had Type C.

The current study revealed that among the 60 children evaluated, 16.6% exhibited some form of hearing loss. Sensorineural hearing loss (SNHL) was observed in 6.6% of the participants, while 10% had conductive hearing loss (CHL). Bilateral SNHL in 75% and bilateral CHL in 83.3% of the affected cases. The hearing impairment was mild in nature, but predominantly affected the high-frequency ranges, with the most affected frequencies being 4000 Hz and 8000 Hz.

Out of 10 patients, 4 (40.0%) had sensorineural hearing loss (SNHL) and 6 (60.0%) had conductive hearing loss (CHL). Three out of the four patients in the SNHL group had high frequency mild hearing loss at 4000 HZ and 8000 HZ, while one patient had low- as well as high-frequency mild hearing loss (26–40 db). Three out of these four patients with SNHL had bilateral hearing loss, while one had unilateral SNHL. Five out six patients with CHL had bilateral hearing loss, all of them had mild hearing loss except one had moderate to severe degree.

Two main groups were identified based on audiometric profile: patients with normal audition as shown in all tests used (pass OAE and PT thresholds ≤ 25 dB), and patients with sensorineural hearing impairment in any of the audiometric tests (fail OAE and PT thresholds > 25 dB at any frequency), while children with conductive hearing loss and children where OAE was inapplicable for them were excluded from the comparative studies.

**Fig. 1:**
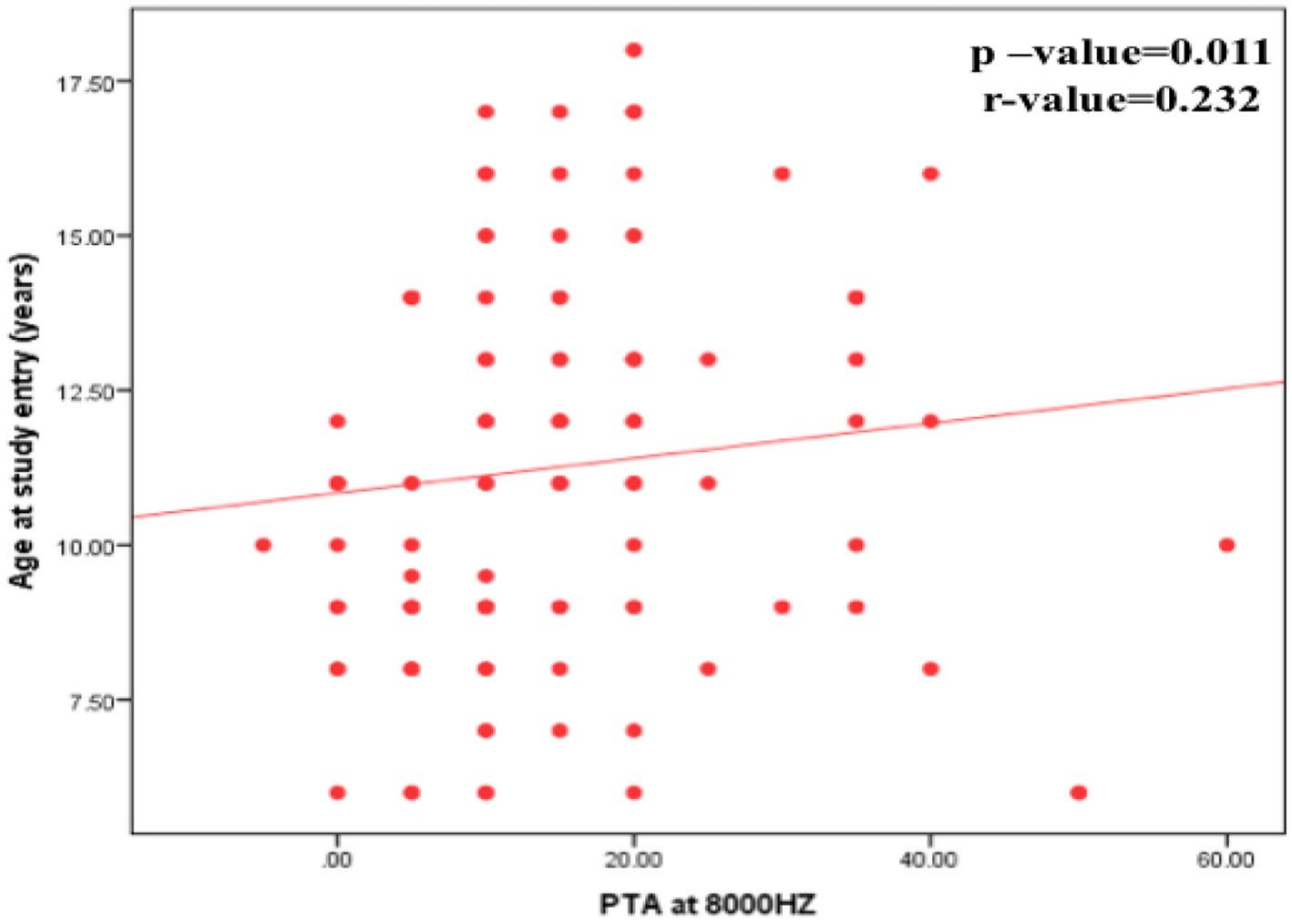
Correlation between PTA at 8000HZ and age of studied patients at the study entry

**Fig. 2:**
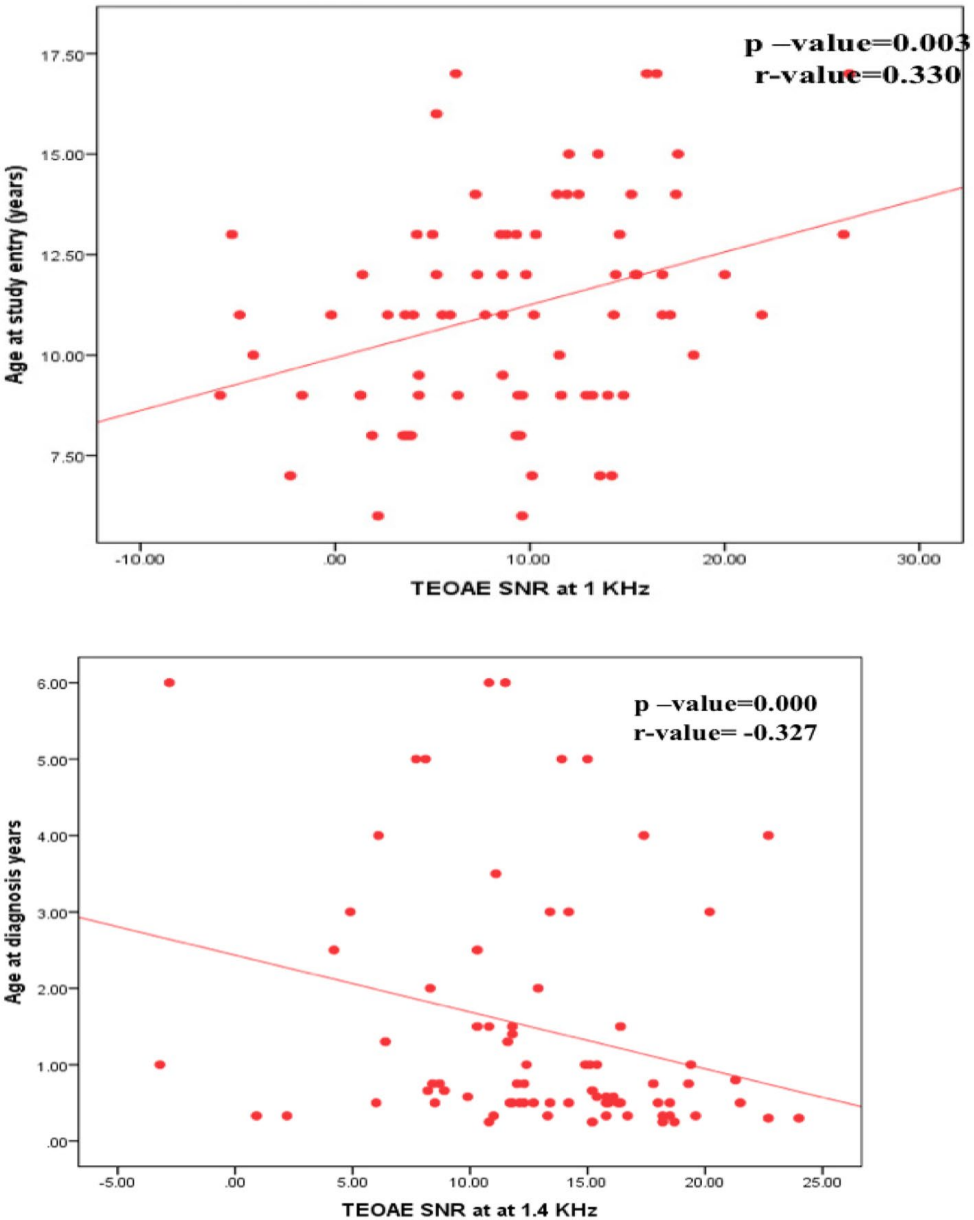
Correlation between TEOAE SNR and age of studied patients at the diagnosis and age at study entry

**Fig. 3:**
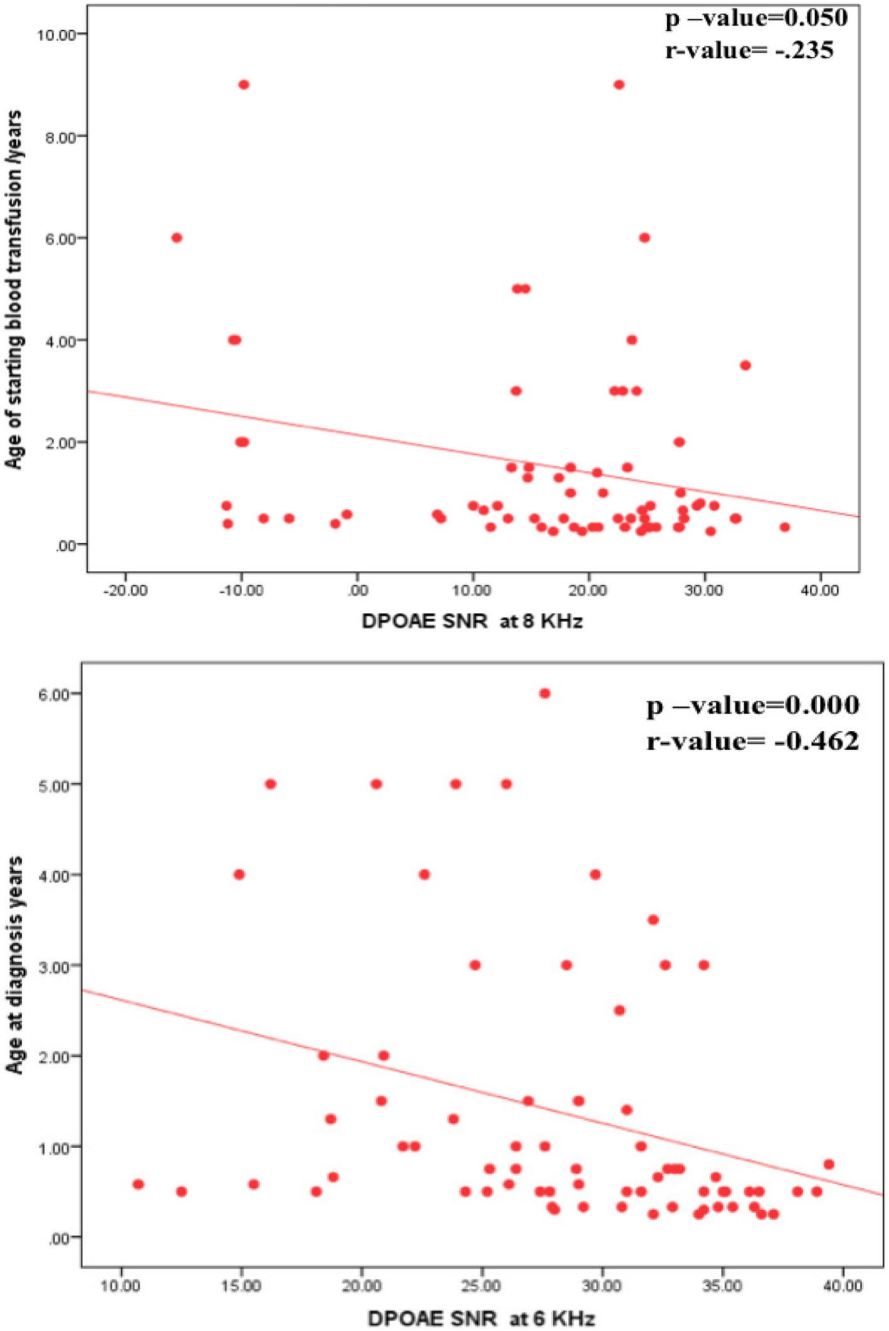
Correlation between DPOAE SNR and age of studied patients at the diagnosis and age at starting blood transfusion

There was no significant difference between studied thalassemia children with and without hearing impairment regarding gender, age at study entry, age at diagnosis and duration of disease (*P* > 0.05) however there was significant correlation between age of the study group, duration of disease and hearing impairment at high frequencies (Figs. 1 and 2), the older the child and the longer the duration of the disease, the worse the hearing thresholds and lower DPOAE SNR at high frequencies (Fig. 3).

Mean haemoglobin was observed to 7.48 ± 0.6 g/dl, 7.85 ± 0.8 g/dl, and 7.8 ± 0.65 g/dl in the SNHL group, CHL group, and normal hearing group, respectively without any significant difference in 3 groups (*p*-value > 0.05). Patients in the SNHL group had an median (IQR) serum ferritin of 533.05 (431.3—1237.3) ng/ml, patients in the CHL group had an median (IQR) serum ferritin of 1284.95 (867.3—1600) ng/ml while patients without any hearing loss had median (IQR) serum ferritin of 1930.6 (1343.3—3271) ng/ml with statistically significant difference in both groups (*P* = 0.028), lower levels found in affected patients. Also our study demonstrate a significant difference between studied thalassemia children with & without hearing impairment regarding Cardiac MRI T2 value (p-value 0.026), higher in affected group, with significant correlation between cardiac MRI T2 value and DPOAEs, the higher the value the worse (lower) SNR at DPOAEs, on the other hand there was no significance regarding liver iron content.

Also our study revealed significant difference between studied thalassemia children with & without hearing impairment regarding age of starting blood transfusion (*p*-value = 0.024),affected patients started blood transfusion at older age. Otherwise, there was no significant difference between the two groups regarding frequency of transfusion or transfusion index (*p*-value > 0.05). Regarding DPAOE results our study revealed a significant correlation between age at starting blood transfusion and DPAOE results, reaching significance at 6000 and 8000 Hz, the younger the age of blood transfusion, the better the SNR in DPOAE. There is also a significant correlation between the frequency of blood transfusion, the more frequent the transfusion, the worse threshold at 1000 and 4000 Hz. (Fig. 3).

There was no statistically significant difference between studied thalassemia children with & without hearing impairment regarding type of chelation drug, dose or duration of treatment, also there is no significant correlation between age at starting chelation, dose and duration of chelation with pure tone audiometry, transient evoked otoacoustic emissions and DPOAE of the studied patients.

## Discussion

Thalassemia is considered a common genetic disorder requiring chronic blood transfusion which results in iron overload with adverse side effects including ototoxicity if patients are not well chelated [17]. Thus, early detection of ototoxicity is important for early intervention [18].

Despite high prevalence of the disease, hearing loss among patients with beta thalassemia remains unclear [4]. Accordingly, this study was conducted and aimed to assess hearing status of TDT children on oral iron chelating agents and to detect other risk factors for hearing impairment in this group of patients.

As regards prevalence of hearing loss in β-Thalassemia Children, the current study revealed that among the 60 children evaluated, 16.6% exhibited some form of hearing loss. Sensorineural hearing loss (SNHL) was observed in 6.6% of the participants, while 10% had conductive hearing loss (CHL). Higher rates were observed using TEOAEs reaching 11.1% of fail response indicating outer hair cell dysfunction and of those with pass TEOAEs 23.2% showed fail response with DPOAEs at high frequencies beyond the measured frequencies from TEOAE, thus indicating that DPOAE was more sensitive to detect early subtle cochlear dysfunction Similarly, The Tiwana et al.,2023 study found a similar hearing loss prevalence of 13% using pure tone audiometry (PTA) but higher rates (16%) when using distortion product otoacoustic emissions (DPOAE) [17]. Also, Osma et al.,2015 found SNHL in 39% of patients using PTA and 22% with DPOAE [16].

Compared to our study, Kong et al.,2014 and Khan et al.,2019 found a higher rate of hearing loss, 57.4 and 45.5% of patients experiencing SNHL, respectively [10, 19]. Also, Galhom et al., 2024 identified a SNHL prevalence of 21.1% in a slightly higher rate than our study [20]. Also a study that included 1422 Iranian TDT patients reported hearing deficit in nearly 27.3% of the patients with sensorineural, conductive, and mixed hearing loss in 10.6%, 14.6%, and 9.1%, respectively [6]. This study found the rate of hearing deficit to be 32.3% although the prevalence rate differed greatly among different studies (0–88.2%) [21]. Relatively lower prevalence in our study could be attributed to the fact that none of these patients were on Deferoxamine which is known to have ototoxic effects as well as small cohort size in our study.

In addition, our study results revealed that the hearing loss predominantly affected both ears, with bilateral SNHL in 75% and bilateral CHL in 83.3% of the hearing impaired cases, all the children with CHL were due to middle ear effusion as confirmed by otoscopic examination and type B tympanogram. The hearing impairment was mild in nature, but predominantly affected the high-frequency ranges with the most affected frequencies being 4000 Hz and 8000 Hz. This is consistent with the findings of Tiwana et al.,2023 who reported that hearing loss primarily affected frequencies of 2000 Hz, 4000 Hz, and 8000 Hz in patients on deferasirox [17]. Osma et al.,2015 study also found that hearing loss was most prevalent in high frequencies, with thresholds affected at 4 kHz, 6 kHz, and 8 kHz [16]. In contrast, Khan et al. (2019) study found that SNHL was more common at 500 Hz and 1000 Hz, suggesting some variation in the frequency range most affected depending on the patient population and study methods [10].

As regards Impact of blood transfusion and Disease Management, one of the critical findings was the relationship between hearing impairment and the timing of blood transfusions. Children who started blood transfusions later in life were more likely to experience hearing issues, and those with lower serum ferritin levels were also at greater risk of hearing loss. Specifically, patients with hearing loss started transfusions at a median age of 1.5 years, compared to 0.66 years for those without hearing issues. There was a statistically significant correlation between starting blood transfusions at an older age and hearing impairment. This suggests that early and regular blood transfusions may have a protective effect against hearing loss in β-thalassemia major patients. several factors have been implicated in the mechanism of SNHL in previous studies, including hypoxia [22]. A previous meta-analysis highlighted that there is a strong correlation between the degree of anemia and SNHL [23]. These findings in literature supports our results that affected patients started transfusion at an older age as this means they suffered from anemia for longer duration before starting transfusion. This explains another risk factor for hearing affection in patients with thalassemia other than the chelation drug.

The study did not find significant correlations between the frequency of transfusions or the volume of transfusions and hearing impairment. This indicates that the timing of the first transfusion may be more critical than the frequency or volume in determining auditory outcomes. A series of studies did not find a statistically significant relationship between blood transfusions and hearing loss, transfusion-related factors were not identified as significant contributors to auditory damage [16–18, 20, 21].

In contrast, Khan et al., 2019 identified a potential link between transfusion practices and hearing loss. The study noted that patients the more frequent blood transfusions were more likely to experience SNHL. Specifically, who had been on transfusions for longer than 60 months were more prone to hearing loss. This suggests that cumulative exposure to iron overload, likely resulting from frequent transfusions, may increase the risk of ototoxicity, particularly in patients undergoing long-term chelation therapy [10].

Our study revealed significant difference in both groups with hearing impairment and without regarding median serum ferritin (*P* = 0.028), lower levels found in affected patients. Development of hearing impairment has been linked to serum ferritin levels although the exact relationship remains controversial. Many studies reported non-significant difference in ferritin level between patients with and without hearing loss [9, 24–26]. Patients with low serum ferritin levels mean that they are well chelated and more compliant on chelation treatment than the other affected group. Hearing impairment in this group of patients with lower serum ferritin could be attributed to oral chelation, however, this is still an indirect relation, and we cannot confirm this. Further studies with larger sample size and longer follow up period of patients may help confirming this. These findings were pointed up in previous studies, where low ferritin levels were considered a risk factor for SNHL [27]. This was attributed to invasive chelation of these patients with DFO [6].

As regards audiological assessments and hearing impairment, the audiological tests conducted in this study, including pure tone audiometry (PTA) and tympanometry, revealed that hearing loss was primarily in the high-frequency range. PTA results showed worse thresholds at higher frequencies in older children and those with longer disease duration, tympanometry tests indicated that the majority of patients (71.2%) had normal middle ear function, while 15.2% had Type C dysfunction, which is typically associated with Eustachian tube problems. This comprehensive audiological approach highlighted the importance of high-frequency hearing assessments especially using Distortion product OAEs in detecting early signs of cochlear affection, similar to our results a long-term audiological follow-up over 20 years using primarily PTA to assess hearing loss in thalassemia patients, the study found that 23.8% of patients developed SNHL, with most cases affecting the high-frequency ranges [18]. In contrast to a large cohort of 198 β-thalassemia major patients the hearing loss observed in this study was predominantly at lower frequencies (500 Hz and 1000 Hz) [10].

Osma et al., 2015 compared both PTA and DPOAE in detecting hearing loss among β-thalassemia patients and found that 39% of patients had SNHL based on PTA, while only 22% were detected using DPOAE [16]. Interestingly, this study reported that PTA was more sensitive than DPOAE in identifying hearing loss, which is in contrast to our study and other studies that typically consider DPOAE more sensitive for detecting early-stage cochlear damage [18, 22]. The study attributed this difference to the fact that DPOAE primarily identifies subclinical damage, while PTA is more likely to detect more established hearing loss. The most affected frequencies in this study were in the high-frequency range (4 kHz, 6 kHz, and 8 kHz), consistent with our research on ototoxicity in thalassemia patients treated with iron chelating agents. The study's reliance on PTA alone raises the possibility that test protocols, population differences, or disease severity could influence the detection of hearing impairment, underscoring the importance of using a range of audiological assessments for a more comprehensive evaluation [24].

There was no significant difference as regards presence of auditory complaints between the groups with and without hearing impairment on audiological testing. Thus, indicating that audiological evaluation should be performed on a regular basis in all children even without presence of any auditory complaints to detect early subtle changes in cochlear functions enabling the early intervention in the form of titrating the dose of chelating agent or switching to alternative agent.

As regards chelation therapy and hearing impairment, most of the children (80%) were on a single agent (deferasirox), while 18.3% were on a combination of deferasirox and deferiprone. The study found no significant correlation between the type, dose, or duration of chelation therapy and the occurrence of hearing impairment. However, the potential for ototoxicity with these drugs, particularly at higher doses and over longer treatment periods, requires regular monitoring and evaluation to ensure early detection of hearing impairments.

The relationship between chelation therapy and hearing impairment in β-thalassemia major patients has been explored across several studies, with varying findings regarding the impact of different chelating agents.

In contrast to our finding, Aldè et al., 2024 found a significant link between prolonged chelation therapy and hearing loss in their 20-year longitudinal study. The study reported that patients who experienced hearing impairment had significantly higher serum ferritin levels and longer durations of chelation therapy compared to those with normal hearing. This suggests that while the chelating agents are essential for managing iron overload, long-term exposure to these agents may increase the risk of sensorineural hearing loss (SNHL). The study, however, did not differentiate between specific chelating agents, instead highlighting the cumulative effect of long-term chelation therapy on auditory health [18]. Similarly, Khan et al., 2019 also found a dose-dependent relationship between deferasirox and hearing loss. The study revealed that patients who were on higher doses of deferasirox (700–1000 mg/day) and had been receiving chelation therapy for longer durations (25–60 months) were more likely to experience SNHL. This finding underscores the potential ototoxicity of deferasirox and suggests that both the dose and duration of chelation therapy play significant roles in increasing the risk of hearing impairment [10].

However, not all studies found such direct correlations did not report a statistically significant relationship between chelation therapy and hearing loss, despite studying a large cohort of patients on deferasirox [17, 20]. This discrepancy may be due to variations in study populations, chelation protocols, or audiological testing methods, but it raises the question of whether certain patients are more susceptible to chelation-induced ototoxicity than others. Similarly, Osma et al. (2015), in their comparison of patients treated with different chelators—deferasirox, deferiprone, and deferoxamine—did not find a statistically significant difference in the incidence of ototoxicity between the various chelating agents. Although 39% of patients exhibited hearing loss using PTA, and 22% were detected with DPOAE, the study concluded that none of the chelators appeared to be more ototoxic than the others. This suggests that the risk of hearing loss may be inherent to iron chelation therapy in general, rather than specific to a particular drug, and that long-term use of any chelator could pose a risk to auditory health [16].

As regards cardiac and hepatic assessments, in our study, cardiac and hepatic assessments were conducted using echocardiography and MRI to evaluate iron deposition in these organs. While 33.3% of the patients had abnormal echocardiographic findings, primarily dilated left ventricles (34.3%) and tricuspid regurgitation (18.7%), most patients (45%) had no cardiac iron overload based on MRI T2* values., there was a statistically significant correlation between worse Distortion Product Otoacoustic Emissions (DPOAEs) and higher cardiac MRI T2* values. Higher Cardiac MRI T2 value means better cardiac health which means that they are better chelated and more compliant on chelation treatment than the other affected group, but this is an indirect relation, we cannot confirm directly through our study that hearing impairment in these patients with higher cardiac MRI T2 values is related to the oral iron chelation. Further studies with larger sample size and longer follow up period of patients may be needed for further clarification.

On the hepatic side, 58.6% of patients had moderate hepatic iron overload, but no significant association was found between cardiac and hepatic iron levels and hearing loss.

Similarly, Aldè et al., 2024 explored the long-term effects of iron chelation therapy and iron overload on the heart and liver. In this study, cardiac MRI and hepatic iron concentration were measured, but no significant differences were found in cardiac T2 values* or liver iron content between patients with and without hearing loss. This suggests that while iron overload in these organs is a common complication of β-thalassemia major, its direct link to hearing loss remains uncertain [18]. Khan et al.,2019 also assessed cardiac and hepatic function, reporting that the mean **s**erum ferritin levels of the study population were extremely high, at over 5000 ng/mL, reflecting significant iron overload. However, the study did not find a direct link between cardiac or hepatic function and hearing loss, although patients with higher serum ferritin levels and longer durations of chelation therapy were more likely to experience SNHL. The study suggests that while serum ferritin is a useful marker of overall iron burden, its impact on hearing might be mediated more through systemic overload and prolonged exposure to chelators, rather than direct damage to cardiac or hepatic tissue [10].

Our study underscores the need for regular audiological monitoring in children with TDT, Given the prevalence of hearing loss, particularly in high-frequency ranges, incorporating pure tone audiometry (PTA) and otoacoustic emissions (OAE) tests into routine evaluations is crucial for early detection and intervention.

Personalized iron chelation therapy should be considered, with regular hearing assessments to detect ototoxicity early. While no direct correlation was found between chelation dosage or duration and hearing loss, careful monitoring allows clinicians to adjust therapy as needed, reducing the risk of permanent auditory damage.

The study also highlights the importance of early blood transfusions to manage iron overload. Starting transfusions earlier may reduce the need for high doses of chelation therapy, potentially decreasing the risk of hearing impairment. Effective iron management can prevent complications in both hearing and overall organ function.

A multidisciplinary approach is essential for managing the complexities of β-thalassemia. Audiologists, haematologists, and other specialists should work together to monitor cardiac, hepatic, and auditory health, given the potential systemic effects of iron overload. Educating patients and families about the risks of chelation therapy and the importance of regular hearing tests is also crucial for early intervention and better outcomes.

### The strength points of this study

A significant strength of our study is the multifaceted approach, which included comprehensive cardiac and hepatic assessments alongside audiological evaluations, providing insights into the broader systemic effects of iron overload in β-thalassemia major patients. The study focus on a pediatric population highlights the importance of early intervention in preventing long-term complications, and the integration of both PTA and tympanometry offers a well-rounded evaluation of hearing health. Additionally, identifying the potential protective effect of early blood transfusion against hearing loss adds valuable information for improving patient care protocols.

### The limitations of this study

One of the key limitations of our study is the sample size, which may limit the generalizability of the findings to a larger population, the lack of baseline audiological evaluation prior to the start of chelation therapy or blood transfusion. Additionally, the cross-sectional design does not allow us to assess long-term effects of iron chelation therapy on hearing loss or detect reversibility of CHL after management of otitis media. The use of more sensitive tests like DPOAE might have provided a more comprehensive evaluation of early cochlear damage. Lastly, the absence of detailed data on dosage variations and duration of chelation therapy may have impacted our ability to identify stronger correlations between treatment and hearing loss.

## Conclusion

The findings of this study suggest that children with transfusion dependent β-thalassemia on oral iron chelation therapy are at risk for cochlear dysfunction particularly affecting high frequencies, particularly high-frequency SNHL. Regular audiological monitoring including both PTA and DPOAE is crucial for early detection and intervention. The correlation between blood transfusion timing, serum ferritin levels, and hearing impairment indicates that optimizing transfusion strategies may reduce the risk of hearing impairments.

## Data Availability

The datasets used and/or analyzed during the current study are available from the corresponding author on reasonable request.

## Abbreviations

BMT: Bone marrow transplant
CBC: Complete blood count
CHL: Conductive hearing loss
dB: Decibel
dBHL: Decibel Hearing Level
dBpeSPL: Decibels peak equivalent sound pressure level
DFO: Deferoxamine
DFX: Deferasirox
DPOAE: Distortion product otoacoustic emissions
Hz: Hertz
KHz: Kilohertz
LIC: Liver Iron Content
OAE: Otoacoustic emission
PTA: Pure tone audiometry
SNHL: Sensorineural hearing loss
TDT: Transfusion Dependent β-thalassemia
TEOAE: Transient evoked otoacoustic emissions
